# Targeting Cancer using Polymeric Nanoparticle mediated Combination Chemotherapy

**DOI:** 10.16966/2470-3206.116

**Published:** 2016-07-04

**Authors:** Aniket Gad, Janel Kydd, Brandon Piel, Prakash Rai

**Affiliations:** 1 Biomedical Engineering and Biotechnology Program, University of Massachusetts, Lowell, USA; 2 Department of Chemical Engineering, University of Massachusetts, Lowell-1 University Ave, USA

**Keywords:** Nanomedicine, Drug delivery, Oncology, Multi-drug resistance, Controlled release, Enhanced permeability and retention, Receptor mediated endocytosis, Biopolymers, Biocompatibility, Biodegradability

## Abstract

Cancer forms exhibiting poor prognosis have been extensively researched for therapeutic solutions. One of the conventional modes of treatment, chemotherapy shows inadequacy in its methodology due to imminent side-effects and acquired drug-resistance by cancer cells. However, advancements in nanotechnology have opened new frontiers to significantly alleviate collateral damage caused by current treatments via innovative delivery techniques, eliminating pitfalls encountered in conventional treatments. Properties like reduced drug-clearance and increased dose efficacy by the enhanced permeability and retention effect deem nanoparticles suitable for this application. Optimization of size, surface charge and surface modifications have provided nanoparticles with stealth properties capable of evading immune responses, thus deeming them as excellent carriers of chemotherapeutic agents. Biocompatible and biodegradable forms of polymers enhance the bioavailability of chemotherapeutic agents, and permit a sustained and time-dependent release of drugs which is a characteristic of their composition, thereby providing a controlled therapeutic approach. Studies conducted *in vitro* and animal models have also demonstrated a synergism in cytotoxicity given the mechanism of action of anticancer drugs when administered in combination providing promising results. Combination therapy has also shown implications in overcoming multiple-drug resistance, which can however be subdued by the adaptable nature of tumor microenvironment. Surface modifications with targeting moieties can therefore feasibly increase nanoparticle uptake by specific receptor-ligand interactions, increasing dose efficacy which can seemingly overcome drug-resistance. This article reviews recent trends and investigations in employing polymeric nanoparticles for effectively delivering combination chemotherapy, and modifications in delivery parameters enhancing dose efficacy, thus validating the potential in this approach for anticancer treatment.

## Background

Many prevalent forms of malignant cancers have accounted for high mortality rates for the past few decades. Although substantial development is achieved in chemotherapeutic treatments, effective diagnosis and treatment of cancer involves careful consideration of the heterogenous tumor microenvironment, an area that is relatively poorly understood. The tumor microenvironment is created in response to the progression of a neoplastic disease state which arises through what is known as the “hallmarks of cancer”. The key hallmarks include sustained proliferative signaling, evasion of growth suppressors, resistance to cell death and subsequent cell immortality, evasion and in some cases even recruitment of the immune system, angiogenesis or blood vessel formation, invasion and metastasis. Tumor cell heterogeneity is a result of inflammation and genomic instability, where a single advantageous mutation exists without repair and further mutations in cell divisions are permitted in the cancerous cell population. The characteristics of reprogrammed cell energy metabolism and evasion of the immune system are also key factors in the formation of the tumor cell microenvironment. The genetic alterations, cell abnormalities, complexities and heterogeneous nature can lead to multi-drug resistance (MDR) by limited access to tumors and nonspecific targeting when using single drugs [1].

**Figure 1:**
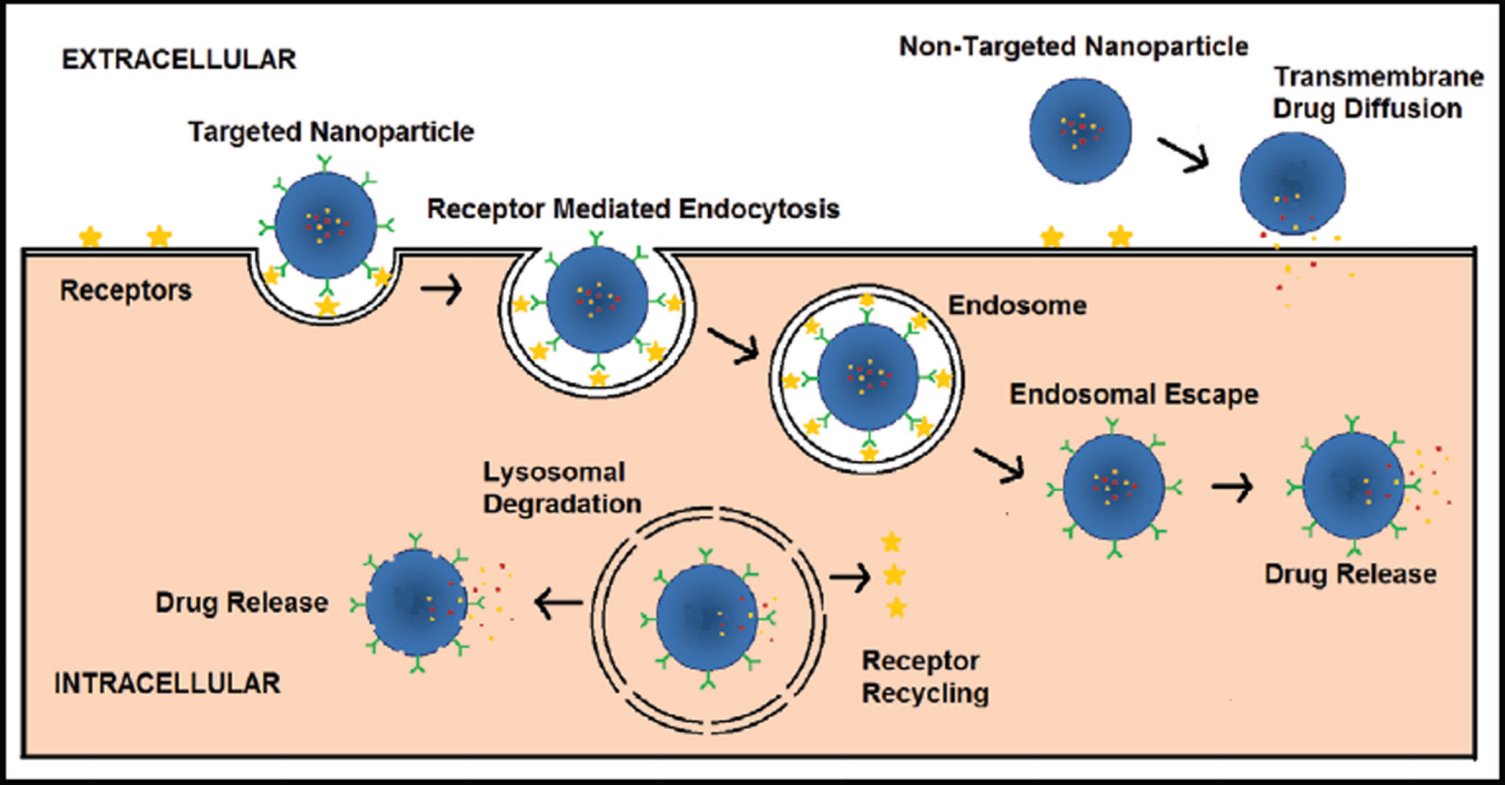
Scheme illustrating differences in drug release and cellular localization for targeted and non-targeted PEGylated polymeric nanoparticles. Targeted nanoparticles are taken up by Receptor mediated Endocytosis while non-targeted nanoparticles may release drug in the extracellular space which then diffuses across the cell membrane.

Commercially available chemotherapeutic agents with established anticancer properties are now being explored using nanotechnology. The advent of nanomedicine has reduced the obstacles encountered in conventional treatments by decreasing drug-related toxicity and MDR, while improving plasma half-life, bioavailability and biodistribution of drugs [2–4]. Nanoparticles facilitate a sustained, controlled and targeted drug delivery method which enhances dose efficacy and reduces side effects. An added advantage is the increase in the drug-uptake by enhanced permeability and retention (EPR) effect which takes advantage of the imperfect tumor vasculature. Moreover, actively targeting nanoparticles to malignant cells via receptor-specific interactions can demonstrate an increased uptake due to receptor mediated endocytosis (RME) (Figure 1). As illustrated in figure 1, non-targeted nanoparticles may be phagocytozed by certain cells or may act as drug depots in the extracellular space and release drug which then may diffuse across cell membranes to the cytosol where most drug targets reside. Well-designed, targeted, polymeric nanomedicines can be internalized via RME and then undergo endosomal escape thereby avoiding destruction in lysosomes due to the harsh environment including low pH and enzymatic degradation. Endosomal escape can thus help release drugs into the cytosol and improve treatment efficacy especially for diseases like cancer.

Recent research has employed combination therapy to target various metabolic and physiological characteristics in cancer cells in order to reduce drug resistance, however pharmacokinetics vary and inconsistent drug uptake within tumor cells and suboptimal drug combination at tumor sites occurs [1–3,5–7]. The maximum tolerated dose (MTD) does not factor in drug synergisms which are affected by drug dosing and scheduling of multiple drugs [2]. The overall therapeutic effects are greater than the additive effect of the individual drugs in synergistic combination drug therapy [2–4,8–12]. Co-delivered drugs can target similar or different pathways and function synergistically to increase efficacy and selectively [4]. The co-encapsulation of drugs with different physicochemical properties, drug loading ratios and sequential drug-release in nanoparticles therefore proves useful in combination therapy [2–4,11–13]. Application of combination therapy via free-drug regimens in clinical trials has exhibited a treatment effect advocating the use of combination drugs over single-drug regimens. Moreover with nanotechnology, considering the degradation characteristics of polymeric nanocarriers and significant differences in release patterns of multiple drugs have shown enhanced synergism in combination therapies [12,14].

One of the commonly used polymers, poly (lactic-co-glycolic acid) (PLGA) is an FDA-approved polymer employed in many biomedical applications due to its excellent biocompatible and malleable properties [15]. Its biocompatible nature prolongs its blood circulation, thereby increasing the plasma half-life of encapsulated drugs in addition to its advantages including high-drug loading capacity favoring hydrophobic drugs, subsequent increased intracellular delivery of drugs and solid matrix protection of the drugs against degradation. Evasion of the mononuclear phagocytic system (MPS) utilizing diblock polymers or polyethylene glycol modified (PEGylated) forms such as PLGA-PEG further enhance the systemic circulation time, allowing a greater uptake of chemotherapeutic agents. The individual block component ratios can be modified to suit a particular application thereby allowing control over the rate of polymer degradation, and hence a desired drug-release profile [15]. Polymeric nanocarriers allow for conjugation of targeting ligands capable of actively enhancing uptake in malignant cells, thereby exploiting the characteristic leaky tumor vasculature allowing selective extravasations of conjugated nanoparticles and longer retention time due to poor lymphatic drainage [1].

Investigations in *in vitro* cultures and animal models have determined aspects of nanoformulations capable of enhancing the efficacy of treatments in clinical settings. Dose efficacy estimations by cytotoxicity assays and assessment of drug-release profiles provide an improved understanding of the treatment mechanism, thus evaluating the potential of polymeric nanoparticles in anticancer applications.

Particle size and surface characteristics are primary features influencing the bioavailabilty of encapsulated chemotherapeutic agents to tumor sites. Recent *in vitro* and animal model studies have highlighted the importance of nanoparticle sizes less than 200 nm accounting for longer systemic circulation time, lower cytotoxicity, greater stability and favorable uptake by the EPR effect [11–13,16–20]. Nanoformulations with relatively larger sizes (<500 nm) are prone to systemic clearance and have demonstrated the need for suitable surface modifications to potentially evade MPS recognition [21]. Conjugation of chemotherapeutic agents and targeting ligands to the polymer backbone has been implemented as an effective approach in optimizing actively-targeted nanoformulations [9]. Size determination of modified nanoparticles by Dynamic Light Scattering (DLS) and Transmission Electron Microscopy (TEM) have indicated minor fluctuations in sizes post surface modifications and drug loading, while still retaining their sizes in the ideal range [18,20]. Surface charge greatly regulates cellular interaction of nanoparticles, with cationic nanoparticles demonstrating a higher cellular uptake as compared to anionic particles [18,21,22]. However, in the case of polymeric nanoparticles positive surface charge has been associated with increased cytotoxicity *in vivo*. Suitable surface modification in several studies to shield cationic groups; for example, the use of PEG has demonstrated reduction in cytotoxic effects due to cationic charges [16]. Particles with a low anionic charge (−20 mV to −40 mV) would present as ideal candidates for *in vivo* application therefore striking a balance between charge related cytotoxicity and uptake [2,18,21,22].

The interdependency of polymer composition and particle characteristics discussed above has been crucial in the development of nanoformulations. Hydrophobic and hydrophilic natures of polymer components influence the drug loading capacity and facilitate conjugation of targeting moieties and chemotherapeutic agents [2], with hydrophobic drugs such as paclitaxel, curcumin, cisplatin and docetaxel displaying high encapsulation in hydrophobic polymer cores via self-assembly over hydrophilic drugs such as gemcitabine, anthracycline and irinotecan. However, alternative approaches such as surface conjugation of drugs and modifications in polymer composition can enable greater encapsulation of such hydrophilic agents [9,18–20,22]. The important aspect in combination chemotherapy however is the synergistic effect of the delivered chemotherapeutic agents. Traditionally limited by poor bioavailability and short plasma-life [8,23], nano-formulations delivering combination chemotherapy provide an improved, controlled and sustained release, thereby showing synergistic effects. Combination therapy has allowed for dynamic re-networking of signalling mechanisms permitting a time and pH-dependent release of drugs providing synergistic effect *in vitro* cultures [3,23].

**Figure 2:**
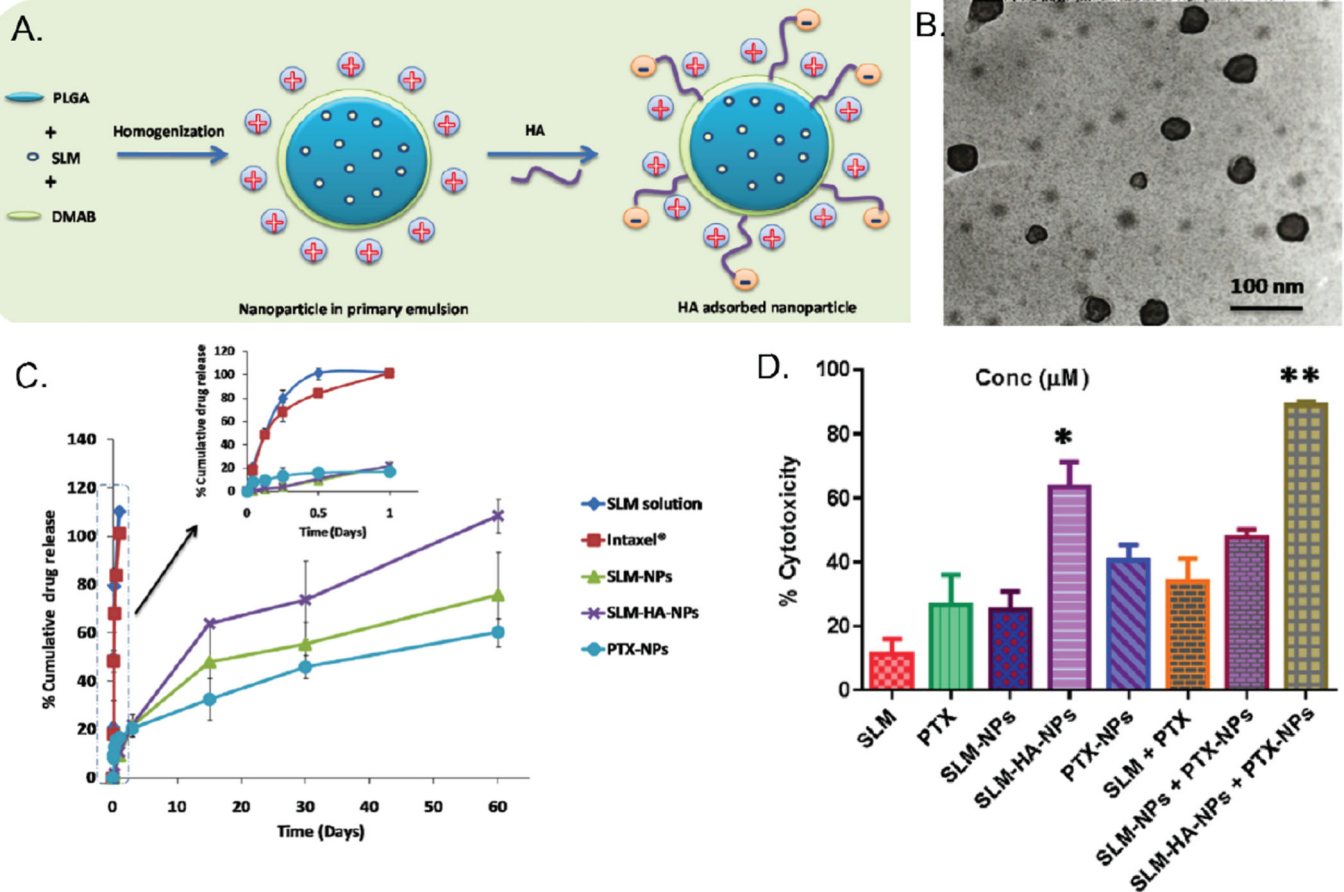
Comparison of Various Paclitaxel and Salinomycin Delivery Vehicles. a) Illustration of SLM-HA-NP. In the presence of diododecyltrimethylammonium bromide (DMAB), the nanoparticle surface becomes positively charged. The addition of hyaluronic acid partially neutralizes the positive charge of the nanoparticle. b) TEM imaging of nanoparticles confirming their size and spherical shape. c) In vitro drug release study. Complete release of SLM and PTX was achieved after 60 days. d) % cytotoxicity of different SLM and PTX formulations, including free drugs, nanoencapsulation, targeted nanoencapsulation, and dual-loaded targeted and non-targeted nanoparticles after 48 h of exposure. Cytotoxicity was determined by MTT assay. Adapted from Muntimadugu et al. [18]

In work presented by Muntimadugu et al., a classic example of synergism due to combination therapy is showcased via targeted PLGA nanoparticles for breast cancer treatment (Figures 2A and 2B). Fairly monodisperse particles with a polydispersity index (PDI) less than 0.3 were synthesized with minor increase in sizes post paclitaxel (PTX) and salinomycin (SLM) loading and surface modification, while still maintaining sizes under 150 nm. Particles displayed a positive surface charge of +50 mV conferred by diododecyltrimethylammonium bromide (DMAB), hyaluronic acid (HA) ligand-modified surface and loading of chemotherapeutic agents. Although a positive surface charge contributed towards a higher particle uptake, this study did not evaluate the cationic charge-related cytotoxicity that would have presented as an issue *in vivo*. Nanoparticles (NPs) displayed high encapsulation of paclitaxel and salinomycin individually given their hydrophobic nature, however attempts to co-encapsulate these agents in a single carrier significantly reduced encapsulation of only paclitaxel. Combination of PTX NPs and HA-targeted SLM NPs demonstrated highest synergistic effect favored by HA targeting and sustained release of these chemotherapeutic agents. SLM-NPs and targeted SLM-HA-NPs showed a complete drug-release over a period of 60 days, with a longer release time in PTX-NPs considering the hydrophobic-hydrophobic interactions of drugs and polymer components (Figure 2C). Nanoformulations demonstrated up to a two fold increase in cytotoxicity when compared to their free-drug counterparts in MCF-7 cells, and a four-fold increase in SLM-HA-NP targeted formulation (Figure 2D). This combination therapy, even though not optimized for co-encapsulation of chemotherapeutic agents, exemplifies the potential of combination therapy using polymeric nanocarriers.

The true value of using polymeric nanoparticles for combination therapy in cancer can be assessed *in vivo* using relevant models of the disease. A study conducted by Shin, et al. illustrated a polymeric micelle based delivery method that employed the use of three medications. Paclitaxel, 17-AAG, and rapamycin were conjugated to a PEG-b-polylactic acid (PLA) copolymer. Since these three drugs are hydrophobic, polymeric micelle conjugation decreases the hydrophobicity of the treatment. It was determined in this study that the three-in-one loading method could deliver these cytotoxic agents safely and effectively to the tumor site. The evidence supporting this included a high tolerance of the drug in FVB albino mice. This study determined that the half-life of the drug was between 1–15 hours, illustrating safe decomposition of the drug *in vivo* [24].

**Figure 3:**
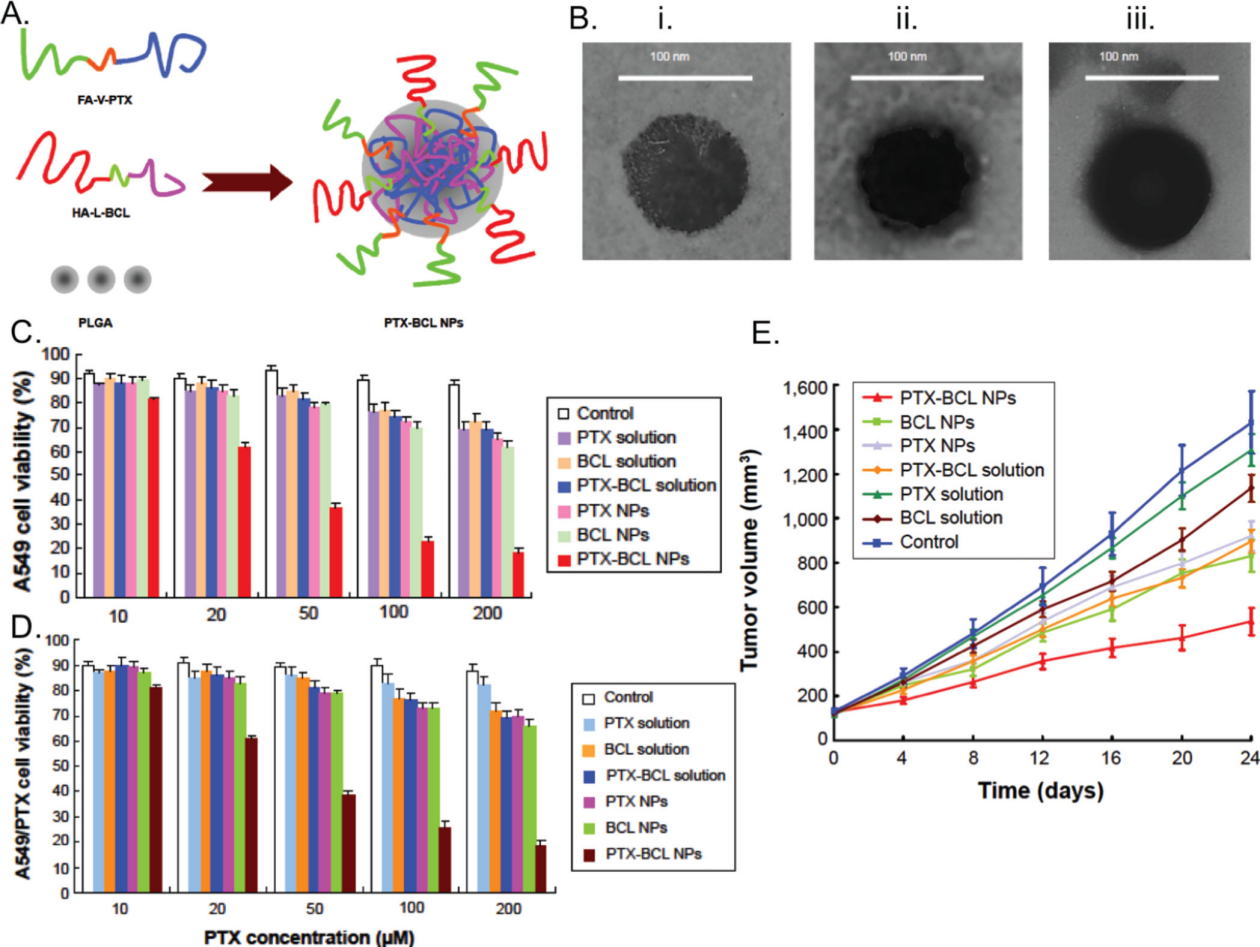
Synthesis, cytotoxicity and effects on tumor volume of paclitaxel and baicalein combination nanoformulation. a) The targeted PTX-BCL NPs synthesis approach is shown using HA and FA targeting ligands which yielded greater than 86% encapsulation for both drugs. b) NP sizes less than 100 nm were obtained by TEM imaging which was favorable for the application. The cytotoxicity of combination NPs was higher than free-drug and single-drug NPs in c) A549 cells and d) paclitaxel-resistant A549 cells observed. e) The lowest tumor growth rate was observed in PTX-BCL NPs compared to free-drug formulations or single-drug NPs. The PTX/BCL ratio was ⅕ (w/w) in PTX-BCL NPs and free drug PTX-BCL solution.

In a study by Wang et al. prodrugs of baicalein (BCL) and paclitaxel (PTX) which contained dual-targeted ligands of folic acid (FA) and hyaluronic acid (HA) were utilized in a prodrug-based nano-drug delivery system (P-N-DDS). Results of this study have been reproduced in figure 3. The P-N-DDS combines two polymer-drug conjugates which each carry single drug agents (Figure 3A). Valine and lysine are used as connections between the drug and the ligands to obtain the prodrug. Amino acid linkers versus poly-ethylene-glycol (PEG) provide the advantage of weaker bonds that allow for faster drug-release. PEG has been associated with lower efficacy than the drug alone. This study used nanoprecipitation to make NPs which had BCL and PTX in the inner core of a PLGA polymer-based NP. These nanoparticles were characterized by TEM (Figure 3B). The synergistic, antitumor effects of combined drug therapy were assessed *in vitro* using human lung cancer A549 cells (Figure 3C) and drug-resistant lung cancer A549/PTX cells (Figure 3D). CD44/CD168 receptors and folate receptors over expressed on lung cancer cells which provided a targeted mechanism for NP drug delivery with HA and FA binding to these receptors, respectively. *In vivo* studies were performed in mice with A549/PTX drug resistant human lung cancer xenograft to determine antitumor efficiency and systemic toxicity. The statistical significance of the results was tested using the two-tailed t-test or one-way analysis of variance, whereby a P value less than 0.05 was considered statistically significant.

PTX-BCL NPs had an average size, PDI, and zeta potential of 91.8 ± 2.3 nm, 0.1 ± 0.03, and 3.3 ± 0.6 mV, respectively. PTX and BCL in the PTX-BCL NPs had an EE value of 91% and 88%, respectively. PDI showed uniformity in the NPs, while the positive zeta potential allowed for increased residence time; cell penetration and internalization of the NPs. High EE values were desirable for *in vitro* cytotoxicity and *in vivo* antitumor efficacy. Cytotoxicity assays were performed *in vitro* using the MTT assay. PTX-BCL NPs showed greater cytotoxicity in A549 cells than other NP formulations or free-drug solution (P<0.05). PTX NPs and BCL NPs also showed greater cytotoxicity than PTX-BCL solution. Combination therapy results for both types of cells *in vitro* showed a pronounced synergistic effect of PTX-BCL when using PTX: BCL ratios of 1:5 and 1:2. A ratio of 1:5 was used *in vivo* in PTX-BCL NPs. *In vivo* studies demonstrated that PTX NPs were less cytotoxic than BCL NPs, possibly due to the suppression of PTX MDR by BCL. Both PTX-BCL solution and PTX-BCL NPs showed better antitumoral effects over PTX alone (Figure 3E). Tumor growth was significantly inhibited by NP formulations compared to free drug solutions. Tumor inhibition was more successful using drug loaded NPs versus free drug solutions. Tumor regression resulted from the use of PTX-BCL NPs as well. Body weight loss was used as an indicator for systemic toxicity (data not shown). No significant weight loss was found with the use of PTX-BCL NPs, while toxicity was observed in PTX solution and PTX-BCL solution treated specimens.

The study noted that future experiments would need to determine optimal doses for anticancer effects and minimal systemic toxicity, as well as applications of the procedure to other types of cancer [14]. The use of positively charged NPs must be taken into consideration, however. A positive zeta potential, although useful in cell membrane penetration and drug uptake, may be hazardous *in vivo* [25]. Cationic NPs are not currently approved by the FDA for clinical use due their enhanced cytotoxicity characteristics. There are known destructive effects on cell membranes caused by cationic NPs, in addition to dose and time dependent hemolytic anemia and pulmonary side effects [25]. Zeta potentials falling between ± 20 mV are desirable and infer electrical stability of the NPs, while small zeta potentials may result in coagulated NPs and less stability [25].

## Conclusions

Polymeric nanocarriers have certain advantages over other modes of drug delivery like free and conjugated drugs. Nanoencapsulation provides a more efficient and stable delivery mechanism of chemotherapeutic agents, especially if those agents are hydrophobic. The ability to fabricate polymeric nanoparticles with sizes under 200 nm and a negative surface charge favors them as carriers in comparison to other encapsulation methods such as liposomes and dendrimers. Dual-loaded particles convey much higher efficacy than combined free-drug solutions as seen in the study by Wang et al. [14]. By controlling particle size, charge, and conjugating targeted ligands to the particle, a drug that evades clearance with tumor target specificity can be created.

As seen in the Muntimadugu et al. [18] and Parhi et al. [2] studies, the addition of a targeted ligand to the nanoparticle surface greatly enhances drug delivery by a factor of 2-fold compared to non-targeted nanoparticles. By choosing which ligands to incorporate into the nanoparticle, the researcher can create a custom delivery mechanism to match the cancer type, ensuring tumor cell specificity. This greater specificity, high blood plasma stability, longer drug-release time, and clearance evasion, offers an improved treatment over chemotherapy alone.

## Future Directions

Polymeric nanoparticles have created many alternative methods of drug loading, as seen in the sections above. Due to the availability of many different types of biopolymers, various medications can be loaded into nanoparticles and then effectively released at the desired target site. Finally, by loading polymeric compounds with multiple drugs to target multiple hallmarks of cancer, more effective treatment methods can be discovered. These nanoparticles can be targeted to deliver drugs at a desired location.

While targeted therapy using NPs is promising in treating neoplastic diseases, there are acquired traits, or hallmarks, that may cause the drug to be ineffective over time because of the complex adaptation of cancerous cells to cellular environmental stresses. Transitory clinical responses have been followed by relapses of disease-state due to the targeting of one capability of the cell and subsequent enabling of another. An example given by Weinberg et al. is the efficacy of vascular endothelial growth factor (VEGF) receptor targeting and antiangiogenesis drugs. Cancer cells may reduce their dependence on one mechanism of adaptation and acquire a new trait, thus increasing the likelihood of drug resistance in the future. Inhibition of angiogenesis has been shown to reduce the size of tumors and cause dormancy of cancer cells, however results have been fleeting. Tumor cell adaptations such as invasion and metastasis may be amplified in response to anti-angiogenesis [26]. Zhao et al. [12] also showed that dual-drug loading of doxorubicin and curcumin by a pH sensitive prodrug allowed high drug loading capacity and release of drug contents within the tumor cell cytoplasma and nuclei. A Schiff ’s base linker that breaks in the acidic environment of the tumor allows for targeted therapy in tumor cells. The aforementioned studies target tumor cells in diverse approaches but similarly strive to achieve ratiometric controls of drug concentrations in order to provide cytotoxic effects on targeted tumor tissue via synergistic drug co-delivery. Biopolymers could be used in similar fashion to achieve controlled release of multiple drugs targeting different hallmarks of cancer for effective cancer treatment.

The future of successful NP use in treating cancer lies in the understanding of genetic factors such as spontaneous and induced mutations (such as in virus associated cancers), the subsequent DNA proofreading and apoptotic signaling pathways, epigenetic markers, micro-RNAs, antibody therapy use in combination with chemotherapy drugs, and heterotypic interactions of different cell types within the body during the various stages of neoplastic disease. The phenotypic differences between normal and cancer cells along with the use of the hallmark traits of cancer will continue to bring more questions and answers as research evolves and methods of detection change [26].

Polymeric nanoparticles are one of the most studied organic strategies for nanomedicine, especially for combination therapy against cancer. Tremendous interest lies in the potential of polymeric nanoparticles to revolutionize modern, personalized cancer medicine. To determine the ideal polymeric nanoplatform for more effective and targeted delivery of drugs, particle size, morphology, polymeric material choice, and processing techniques are all going to remain major research areas of interest. Applications of polymeric nanoparticles include drug delivery via techniques such as conjugation and entrapment of drugs or prodrugs, stimuli-responsive systems, imaging contrast agents, and theranostics. Issues of scale-up in manufacturing and poorly defined, regulatory considerations continue to remain the major challenges in the clinical development of polymeric nanoparticles. However, with increased collaborations between academia and industry, learning from past regulatory successes and the development of better *in vitro* and *in vivo* models continued success in the field is guaranteed.
